# Sensing the Generation of Intracellular Free Electrons Using the Inactive Catalytic Subunit of Cytochrome P450s as a Sink

**DOI:** 10.3390/s20144050

**Published:** 2020-07-21

**Authors:** Damilare D. Akintade, Bhabatosh Chaudhuri

**Affiliations:** 1 School of Life Sciences, Medical School, University of Nottingham, Nottingham NG7 2UH, UK; 2 Leicester School of Pharmacy, De Montfort University, Leicester LE1 9BH, UK; BChaudhuri@dmu.ac.uk

**Keywords:** CYP activation, biosensor, mitochondria and CYP, yeast CYP expression, free electron and CYP

## Abstract

Cytochrome P450 reductase (CPR) abstracts electrons from Nicotinamide adenine dinucleotide phosphate H (NADPH), transferring them to an active Cytochrome P450 (CYP) site to provide a functional CYP. In the present study, a yeast strain was genetically engineered to delete the endogenous CPR gene. A human CYP expressed in a CPR-null (yRD^−^) strain was inactive. It was queried if Bax—which induces apoptosis in yeast and human cells by generating reactive oxygen species (ROS)—substituted for the absence of CPR. Since Bax-generated ROS stems from an initial release of electrons, is it possible for these released electrons to be captured by an inactive CYP to make it active once again? In this study, yeast cells that did not contain any CPR activity (i.e., because the yeasts’ CPR gene was completely deleted) were used to show that (a) human CYPs produced within CPR-null (yRD-) yeast cells were inactive and (b) low levels of the pro-apoptotic human Bax protein could activate inactive human CYPs within this yeast cells. Surprisingly, Bax activated three inactive CYP proteins, confirming that it could compensate for CPR’s absence within yeast cells. These findings could be useful in research, development of bioassays, bioreactors, biosensors, and disease diagnosis, among others.

## 1. Introduction

CYP450 catalyzes xenobiotic metabolism phase I reactions that mostly occur via oxidation reactions for both exogenous compounds and endogenous substrates [1]. However, CYP can also catalyze reduction reactions. CYPs can convert most prodrug to active species but deactivate several drugs used clinically [1]. Functional cytochrome P450 (CYP450) proteins are enzymes that take part in a diverse number of biochemical reactions in different organisms [2]. Over 90% of drugs currently in the market are acted on by CYP enzymes [3]. The supply of electron to the heme iron is required for the catalytic cycle in the presence of oxygen [3]. Deletion coordination of the CYP450 to Fe^3+^ occurs via a thiolate, which is contributed by the –SH of cysteine present in the CYP450 protein. CYP450s are known as haem-thiolate proteins [4]. CYP450 (CYP) enzymes are present in all organisms and belong to a superfamily of proteins [5]. They are the main catalyst involved in the biotransformation of a wide range of chemical compounds that act as substrates to the CYP450 enzymes. The compounds could be either exogenous (xenobiotics) or endogenous to the human body. The biotransformation mediated by CYP450 enzymes occurs through oxidation. One of the reactions that CYP450 enzymes perform on their substrates is the hydroxylation of unreactive carbon atoms [4,6,7].

CYP450 enzymes can only catalyze different substrates in the presence of an NADPH-reductase, which belongs to a family of highly conserved diflavin enzymes [8,9]. All reactions performed by CYP450-CYP reductase binary complexes are NADPH-dependent. These NADPH-reductase dependent CYP450 enzymes are capable of metabolizing most human-made synthetic chemicals. Metabolism (i.e., CYP-mediated biotransformation) involves more than 60 distinct classes of chemical reactions. Compared to standard chemical reactions, CYP-mediated biotransformation reactions are classified as ‘green’ since they do not involve extreme temperatures and pressures. All CYP-mediated chemical reactions can be conducted at a body temperature of 37 °C. It is important to emphasize that these oxidative reactions always occur in close concert with an NADPH-dependent cytochrome P450 reductase CPR [8,9], as discussed below.

### 1.1. Cytochrome P450 Activation through Reductase

CYP450 NADPH-reductase (CPR) is always at the centre of the initial activation of CYP450 proteins so that CYP450s can manifest their enzymatic functionality [6]. Without CPR, CYP450s cannot be activated. Usually, a monooxygenation reaction catalyzed by a CYP450 requires the sequential input of two electrons and protons into the system [6]. This gives the go-ahead for the insertion of activated oxygen into the substrate and the production of a hydroxylated product [6]. CPR belongs to a family of diflavin oxidoreductases that contain an equivalent of flavin adenine dinucleotide (FAD) and flavin mononucleotide (FMN) per polypeptide chain [10,11,12]. A reducing counterpart is transferred from NADPH by CPR through FAD and FMN in a redox reaction [13]. In other words, CPR transfers reducing equivalents from NADPH via FAD and FMN cofactors to CYP450s, the redox partner proteins. CPR enhances the movement of reducing counterparts from NADPH, which donates two electrons, first to the metallic cofactor of the partially bound CYP450 protein, which then accepts the second electron [13]. The role of the CYP450 reductase (CPR) is fundamental to the activity of CYP450s.

The CYP450-CPR reaction produces reactive oxygen species (ROS), superoxide, which is a reduced form of oxygen, and hydrogen peroxide due to the uncoupling of CYP450-CPR reaction [14,15,16,17]. Hence, diffused reactive oxygen species (DROS) have been credited as an uncoupling event that may occur at the centre of haem [14,16,17,18]. Because uncoupling results in the loss of NADPH redox equivalents, it is believed to be an uneconomical process [19]. Hypothetically, an equal number of peroxide molecules or hydroxylated products will be produced by the number of molecules of NADPH, but the total amounts of DROS and hydroxylated product produced are usually less than the amount of NADPH consumed. The imbalance is as a result of water being part of the products [20]. The above makes clear that the catalytic property of CYP450s requires the involvement of CPR and an electron donor (i.e., NADPH), which might restrict the routine use of CYP450s for purposes of high-throughput screening, i.e., CPR as toxic and NADPH as highly unstable. Active mitochondrial cytochrome P450 (CYP) complexes are made up of three different proteins: adrenodoxin, adrenodoxin reductase, and mitochondrial CYP enzyme, all of which have essential parts in steroidal syntheses. They are involved in catalyzing stereo- and regio-specific steroid hydroxylation reactions. Mitochondrial CYP enzymes are also responsible for mitochondrial ROS production and could have a vital role in the initiation of mitochondrial apoptosis [21]. ROS is also generated through microsomal metabolism, particularly during phagocytic respiratory bursts [22], when super anions react directly with proteins [23]. In a bid to create alternative electron supply for CYP activation, the electrochemical medium was explored, as discussed in Section 1.2 below.

### 1.2. Electrochemical Activation of CYP450

The past two decades have seen an increased interest in creating an alternative means of supplying electrons for the activation of CYP450 proteins. Initially, there were investigations on the effectiveness of electrochemical reductions in the presence of mediators [24]. Later, others wondered if various forms of electrodes, in the absence of mediators, would be directly useful in the processes involved in electrochemical reduction [25]. Achieving electron transfer directly, using electrodes, to CYP450 proteins to perform diverse forms of redox reactions is currently of intense interest since there could be applications of such systems in bioreactors, bioassays, and biosensor development. Electrode-mediated activation of CYP450s could be used with a certain level of control by varying the potential of the electrode with the aid of reducing and oxidizing reagents [26].

Many of the electrochemical activations of CYP450 proteins have made use of immobilization of CYP450s, on electrodes, to make them transiently electroactive. However, attention has not been given to the CYP450 conformation when immobilized on electrodes. There is the possibility of structural changes occurring in CYP450s during immobilization. Hence, immobilization has been suspected of having a role in CYP450s’ catalytic characteristics and properties [27]. There have been suggestions that conformational changes in CYP450s, as a result of immobilization, could cause the formation of cytochrome CYP420, which is an inactive cytochrome species. CYP420 proteins show a peak at 420 nm in dithionite (CO) difference spectra. Formation of CYP420 would indicate the addition of a proton (H^+^) to the thiolate ligand at the axis and disturbance of the electronic balance of the haem environment [27]. It has been reported that the formation of CYP420 could also be induced by rising temperature or pressure, contact with neutral salts and detergents, presence of organic solvents, and elevation or lowering of pH of the buffers in which membrane-bound CYP450s are stored [27]. There seem to be some challenges when using the electrode’s electron supply to develop an efficient system of electron supply conditions.

### 1.3. Development of Efficient CYP450 Activation

Liquid chromatography-mass spectrometry (LC-MS) appears to be the gold standard for studying CYP450 metabolites. However, the output is limited due to the number of samples that can be processed at a time and the time consumed in analyzing individual samples. Assays based on fluorescence have a high throughput, and although they provide an indirect measure of CYP450 activity, they are widely used [27,28]. As mentioned earlier, in biological systems involving isolated microsomal CYP450s (i.e., membrane-bound CYP450s isolated from whole cells), electrons are transferred to CYP450s by the electron donor’s NADPH or NADH. This is made possible by mediators that aid electron transfer: FAD and FMN. In reality, NADPH and NADH are quickly exhausted during a CYP450-mediated reaction. An alternative way of sustaining the supply of electrons is an electrochemical reduction, which uses electrodes as the source of electrons. The electrochemical methods for CYP450 activation have mostly been limited to anaerobic studies, where a potential attributed to the reduction of CYP450s was obtained with or without substrates [26].

Moreover, the kind of surfactant film used on the electrodes matters; unfortunately, some can prevent strong interactions between CYP450s and a substrate. More importantly, surfactant molecules can occupy the substrate-binding cavity together with the haem active site [26]. The proposition of electrochemical reduction of CYP450s has not made much progress until now, mainly because microsomal CYP450 enzymes are isolated from different cell systems and inherently unstable at room temperature, being stable only at −80 °C. Moreover, the sudden rush of electrons, produced by an electrode, which is more than what happens within living cells, may not be appropriate for CYP450 activation.

The current study investigated the use of low levels of Bax-generated electrons for the activation of inactive CYP1 family of proteins (CYP1A1, CYP1A2 and CYP1B1) under conditions where Bax did not kill yeast cells. The supply of electrons in the heme iron was vital for the catalytic cycle and the development of biosensor development in the cells was crucial in the advancement of an efficient biocatalysts coupling system. This study was an essential step for developing CYP based sensors.

## 2. Materials and Methods

### 2.1. Yeast Strains

The yeast strain W303-1A Mata (ATCC #208352) (yRD^+^) is auxotrophic for the genes *ADE2*, *HIS3*, *LEU2*, *TRP1*, and *URA3*. New yeast strains were derived from this strain by transforming integrative and episomal plasmids (Supporting Information, Sections S1 and S2), which would express Bax from a *GAL1* promoter or CYP 1A1, 1B1, and 1A2 from an ADH promoter (Supporting Information, Figures S1 and S2). yRD^−^ yeast strain was derived by disrupting the CYP reductase (yRD) (CRP) gene in yRD^+^ yeast strain. To disrupt the yRD gene in S. cerevisiae, the plasmid pAUR101/ΔyRD (Supporting information, Figure S4D) was linearized by cutting the unique SwaI site. The resulting linearized DNA was then introduced into the yRD^+^ yeast strain. The integrants were selected on SD plates containing 0.5 µg/µL of aureobasidin (Supporting information, Section S3) [29,30].

### 2.2. Yeast Transformation

Plasmids bearing Bax and CYP gene expression cassettes under control of either the galactose-inducible *GAL1* promoter or Ethanol-inducible promoter (*GAL1*p/*ADHp*; see Supporting Information, Figures S1 and S2) were used for genomic integration/transformation at the *LEU2* chromosomal locus and *URA3* of the W303 yeast strain (yRD^−^). The integrative transformation was carried out using a published protocol [31]. Similarly, the yRD^+^ yeast strain was transformed with episomal plasmids bearing only the *ADH*p-CYPs expression cassettes on a *URA3* auxotrophic marker.

### 2.3. Detection of ROS with Dihydroethidium

We used an AAT Bioquest Fluorimetric Intracellular Total ROS Activity Assay Kit (#22901) to measure ROS. Experiments were performed using a published protocol [32].

### 2.4. Live Cell Assay

The live-cell assay was carried out using standard protocols [33]. CYP1A2 enzymatic activity in yeast cells was determined using 3-cyano-7-ethoxycoumarin (CEC) (final concentration of 16 µM) was the substrate. CYP1A1 and CYP1B1 activities in yeast strains were determined using 7-ethoxyresorufin (7-ER) as the substrate (final concentration of 5 µM).

### 2.5. Determination of IC_50_

IC_50_ was carried out using standard protocols [33], cytochrome P450 inhibitors Alpha-naphthoflavone (α-Naphthoflavone; ANF) (ranges from 0.00001 to 3 µM) for 1A1 and 1B1, and Furafylline (ranges from 0.1 to 100 µM) for 1A2.

### 2.6. Western Blotting

Western blotting was carried out via standard protocols [34], using primary antibodies specific to α-syn (Proteintech, Rosemont, IL, USA, #10842-1-AP) or β-actin (Proteintech; #60008-1-Ig).

## 3. Results

### 3.1. Comparing the Activities of CYPs Expression, Driven by Three Different Promoters, in yRD^+^ and yRD^−^ Yeast Cells

**Figure 1:**
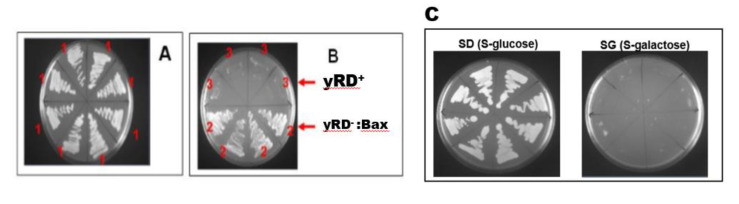
A yeast extract peptone dextrose (YPD) plate (**A**) and (**B**) containing the antibiotic aureobasidin selects for yRD^−^ (**1**) aureobasidin-resistant cells (**A**), while plate B shows four independent sub-clones of yRD^+^ (**3**) and yRD^−^:: Bax (**2**), which were streaked out on the plate. (**C**) Growth of Bax containing yeast cells (yRD^−^:: Bax) on minimal medium (S) agar plates, SD (containing dextrose/glucose) and SG (containing galactose). Cells grow on glucose but do not grow on galactose.

The yRD^−^ yeast strain was resistant to antibiotic aureobasidin. Figure 1A,B shows a non-selective agar plate that contained a full (complete) YPD medium with aureobasidin (0.5 µg of aureobasidin per ml of medium). The yRD^−^ cells contained the aureobasidin A-resistance gene (Supporting information, Section S3), which grew on the plate containing aureobasidin, while the parent yRD^+^ cells did not because they did not have the aureobasidin A-resistance gene. The observations in Figure 1A,B confirmed the disruption of yeast’s endogenous CPR gene (yRD) with a functional aureobasidin A-resistance gene. Before each experiment, CPR-null yRD^−^ cells were selected on aureobasidin-containing YPD plates to continuously ensure that the yRD^−^ cells indeed were devoid of CPR activity.

**Figure 2:**
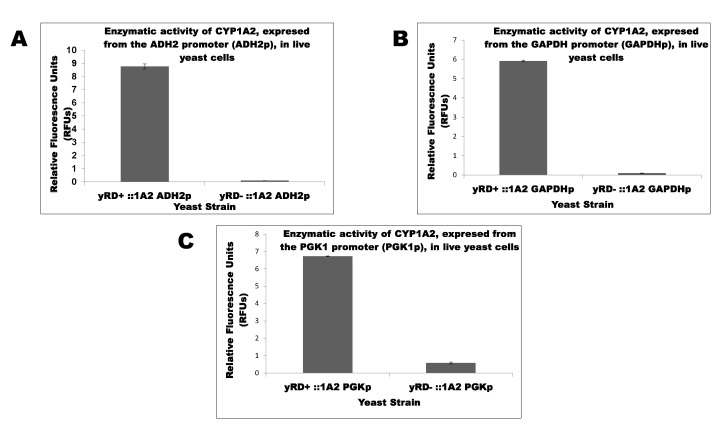
(**A**). Determination of CYP1A2 enzymatic activity in yRD^+^ and yRD^−^ yeast cells using 3-cyano-7-ethoxycoumarin (CEC) as a substrate. CYP1A2 gene expression is driven by the ADH2 promoter (ADH2p). The results show that the CPR (in this case, the yeast reductase, yRD) is essential for CYP450 activity. (**B**) Determination of CYP1A2 enzymatic activity in yRD^+^:: GAPDH-1A2 and yRD^−^:: GAPDH-1A2 yeast cells using 3-cyano-7-ethoxycoumarin (CEC) as a substrate. CYP1A2 gene expression is driven by the GAPDH promoter (GAPDHp). The results show that a CPR (i.e., yRD) is essential for CYP450 activity. (**C**). Determination of CYP1A2 enzymatic activity in yRD^+^:: PGK1-1A2 and yRD^−^:: PGK1-1A2 yeast cells using 3-cyano-7-ethoxycoumarin (CEC) as a substrate. CYP1A2 gene expression is driven by the PGK1 promoter (PGK1p). The results show that a CPR (i.e., yRD) is essential for CYP450 activity.

Initially, yeast strain lacked the endogenous yRD gene (i.e., the yeast CPR) and was transformed with three episomal plasmids (which replicated extra-chromosomally) to the human CYP1A2 gene driven by (a) ADH2, (b) GAPDH, and (c) PGK1 promoters, which had been isolated from the yeast genes alcohol dehydrogenase 2 (ADH2), glyceraldehyde-3-phosphate dehydrogenase (GAPDH), and Phosphoglycerate Kinase 1 (PGK1), respectively. Transformations resulted in the following strains: yRD^−^:: ADH2p-1A2, yRD^−^:: GAPDHp-1A2, and yRD^−^:: PGK1p-1A2. Similarly, the yRD^+^ yeast strain was transformed with the same three plasmids, obtaining the following strains: yRD^+^:: ADH2p-1A2, yRD^+^:: GAPDHp-1A2, and yRD^+^:: PGK1p-1A2. Assays were performed on live cells from the above six strains to monitor the effects of CPR disruption on the activity of human CYP1A2 enzyme; the bar charts in Figure 2A–C show the results.

yRD^−^ cells were used to integrate a copy of the Bax gene expression cassette in yRD^−^ cells’ *LEU2* chromosomal locus to obtain the strain yRD^−^:: Bax(*LEU2*). The resultant cells should also be resistant to aureobasidin. Figure 1B,C shows the full expression of Bax in a galactose medium.

### 3.2. Measurement of CYP1A1, CYP1A2 and CYP1B1 Enzyme Activities within Live Cells, Enzyme Expression Being Driven by the ADH2 Promoter

**Figure 3:**
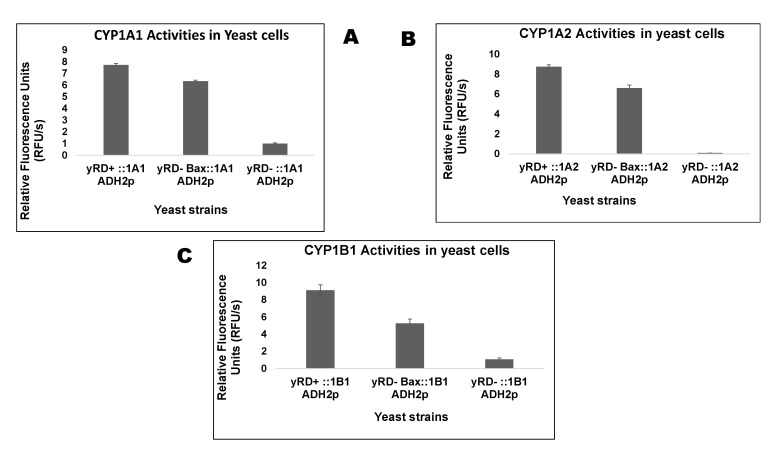
(**A**) Measurement of CYP1A1 activities in yeast strains, yRD^+^ (+CPR), yRD^−^:: Bax (–CPR), and yRD^−^ (–CPR), using 7-ethoxyresorufin (7-ER) as a substrate. CYP1A1 expression, in all three strains, is driven by the ethanol-inducible ADH2 promoter, whereas Bax expression is driven by the GAL1 promoter which is de-repressed in ethanol. The bar charts show that very low levels of Bax expression can indeed substitute for the absence of CPR (i.e., yeast CPR, yRD), which is essential for CYP activity. A between-group ANOVA test identified significant differences (*p* < 0.05) between strains, in all four comparative analyses performed. (**B**) Measurement of CYP1A2 activities in yeast strains, yRD^+^ (+CPR), yRD^−^:: Bax (–CPR) and yRD^−^ (–CPR), using 3-cyano-7-ethoxycoumarin (CEC) as a substrate. CYP1A2 expression, in all three strains, is driven by the ethanol-inducible ADH2 promoter, whereas Bax expression is driven by the GAL1 promoter which is de-repressed in ethanol. The bar charts show that very low levels of Bax expression can substitute for the absence of CPR (i.e., yeast CPR, yRD), which is essential for CYP activity. A between-group ANOVA test identified significant differences (*p* < 0.05) between strains, in all four comparative analyses performed. (**C**) Measurement of CYP1B1 activities in yeast strains, yRD+ (+CPR), yRD-:: Bax (–CPR) and yRD- (–CPR), using 7-ethoxyresorufin (7-ER) as a substrate. CYP1B1 expression, in all three strains, is driven by the ethanol-inducible ADH2 promoter, whereas Bax expression is driven by the GAL1 promoter which is de-repressed in ethanol. The Bar Charts show that very low levels of Bax expression can indeed substitute for the absence of CPR (i.e., yeast CPR, yRD), which is essential for CYP activity. A between-group ANOVA test identified significant differences (*p* < 0.05) between strains, in all four comparative analyses performed.

The three enzymes of the CYP1 family were expressed in yeast using the ADH2 promoter (ADH2p). Like the GAL1 promoter (GAL1p), which drove the expression of human Bax, ADH2p was repressed in glucose. Yeast cells grown in glucose and during growth glucose was converted to its metabolite, i.e., ethanol, which induced the ADH2p. In ethanol, GAL1p was de-repressed, implying that only a shallow level of Bax was produced in ethanol. Biochemical assays were used to measure cytochrome P450 (CYP) activity within live cells using pro-fluorescent substrates that were converted to fluorescent molecules by CYP enzymes via a de-alkylation reaction. Figure 3 shows the live-cell assay of CYP 1A1, 1A2 and 1B1 in the three yeast strains.

The integrating plasmid pRS305/GAL1p-h_Bax-MS (Supplementary Information, Figure S1) had been used to integrate the Bax expression cassette at the *LEU2* locus of the yeast strain yRD^−^ via homologous recombination. The GAL1 promoter controlled Bax gene expression. The resultant strain yRD^−^:: Bax(*LEU2*) (Figure 1C) was transformed with yeast episomal plasmids with a cytochrome P450 gene. The expectation was that on overnight incubation, glucose would be completely converted to ethanol, which allowed for a (a) full-blown induction of CYP1A2 gene expression under the control of the ADH2 promoter and (b) very low levels of Bax expression since the GAL1 promoter (GAL1p), which is fully repressed in glucose, underwent de-repression in the presence of ethanol. Full induction occurred only in the presence of galactose.

### 3.3. Measurement of ROS in yRD^+^, yRD^−^:: Bax and yRD^−^ Cells Expressing CYPs

**Figure 4:**
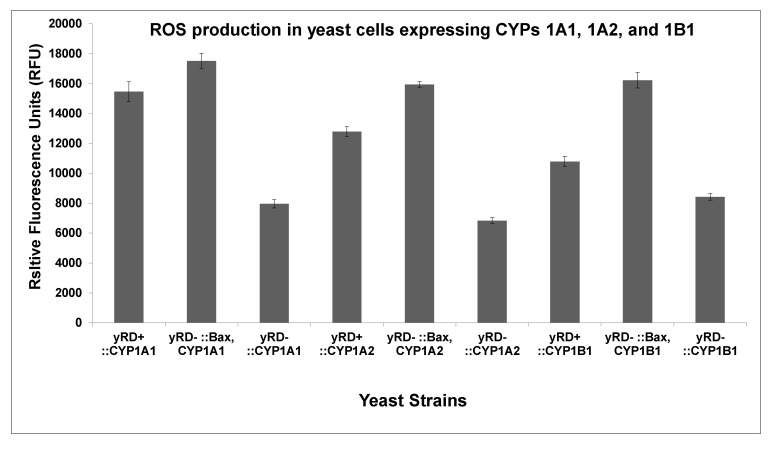
Measurement of ROS production in yeast strains, yRD^+^ (+CPR), yRD^−^:: Bax (–CPR), and yRD^−^ (–CPR), expressing CYP proteins. CYP expression is driven by ethanol-inducible ADH2 promoter while Bax expression is driven by the GAL1 promoter, which is de-repressed in ethanol. ROS production in the three strains expressing CYP1A1, CYP1A2, and CYP1B1. The bar charts show that ROS production was higher in the strains containing Bax and no CPR than the strains that contained CPR, the strains that contained neither CPR nor Bax had the lowest ROS. The significance of ROS production in yeast cells was determined using between-groups ANOVA based on a *p* < 0.05. There was a significant difference (*p* < 0.05) between yRD^+^ (+CPR):: CYP and yRD^−^ (–CPR):: Bax. CYP strains on the one hand and yRD^−^ (–CPR):: CYP contained no CYPs. The ROS production was not significant (*p* > 0.05) between the yRD^+^ (+CPR):: CYP strains and yRD^−^ (–CPR):: Bax, CYP strains.

Figure 4 shows the measurement of ROS production in the three yeast strains of each CYP. The strains carrying the Bax gene produced more ROS compare to the other two strains for each CYP.

### 3.4. Determination of IC50 Values of Known Inhibitors of CYP1A1, CYP1A2, and CYP1B1 in the Strains yRD^+^ and yRD^−^:: Bax and Western Blots to Confirm the Co-expression of CYPs and Bax within Yeast Cells in Ethanol

IC_50_ values of known inhibitors were determined to investigate if the active site geometry of a CYP in the absence of CPR, yet the presence of Bax was the same as the active site conformation in the presence of CPR. To transfer electrons, a CPR had to be in close vicinity of a human CYP, a prerequisite for endoplasmic reticular membranes. Bax, however, was localized on mitochondrial membranes during the apoptosis process.

**Figure 5:**
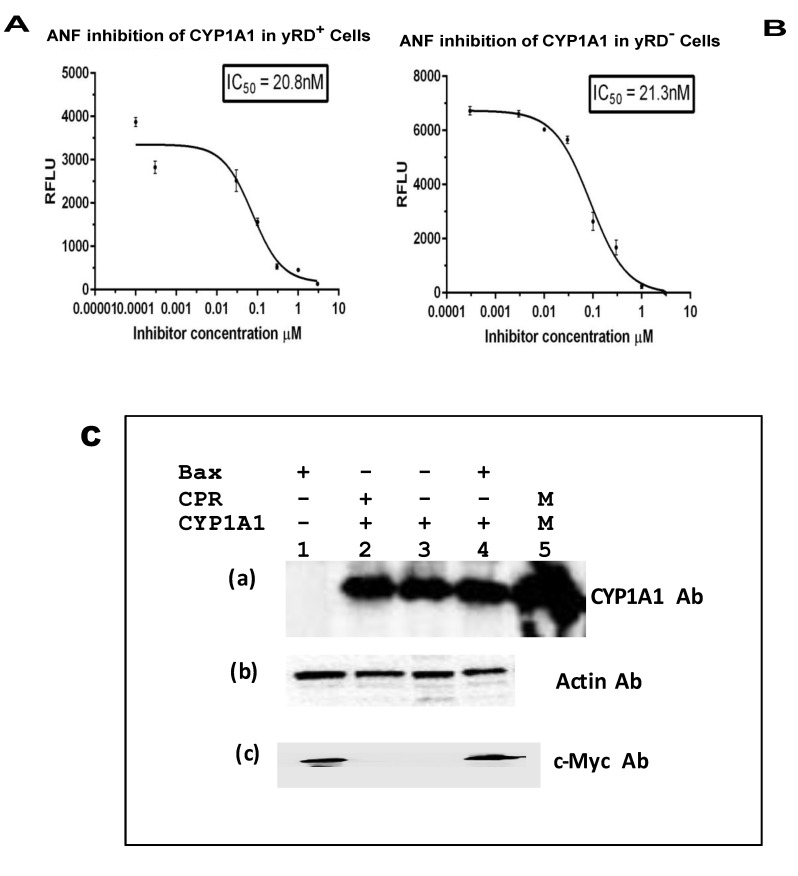
IC_50_ of α-naphthoflavone (ANF) for the inhibition of CYP1A1 enzyme expressed within yRD^+^ and yRD^−^:: Bax cells. ANF’s IC_50_ for inhibition of CYP1A1 enzyme that was expressed in live cells. The cells that were used were from strains (**A**) yRD^+^ (+CPR) and (**B**) yRD^−^:: Bax (–CPR). CYP1A1 enzyme expression was driven by the ethanol-inducible *ADH2* promoter (ADH2p). In yRD^−^ cells, Bax expression was driven by the *GAL1* promoter (GAL1p), which was de-repressed in ethanol. Recombinant cells were grown for 18 h in YPD liquid medium containing glucose, allowing for around 6 h of induction and de-repression of ADH2p and GAL1p in ethanol (it takes ~12 h for glucose to be utilized and entirely converted to ethanol). Cells were harvested, washed in PBS-saline. The assay included 1 × 10^7^ washed cells and 7-ethoxyresorufin (7-ER) as substrates. The *x*-axis represents the values of the concentration of ANF in µM (ranges from 0.00001 to 3 µM) that were incubated with cells for 10 min, the *y*-axis relative fluorescence units of the fluorescent product formed. The IC_50_ values were calculated using the GraphPad Prism software. T. ANF’s IC_50_ for inhibition of CYP1A1 Supersomes (insect cell-derived CYP1A1-bearing microsomes; Corning-Gentest, Corning, NY, USA) is 18 nM [35]. (**C**) Western Blotting of lysates from yRD^+^, yRD^−^, and yRD-:: Bax cells expressing CYP1A1. Yeast cell lysates (5 µg of total cellular protein for monitoring CYP1A1 and actin; 50 µg for Bax) fractionated on SDS/PAGE and probed with antibodies to CYP, Bax and actin proteins after Western blotting. (**a**) Cell lysates probed with a CYP1A1 antibody (Proteintech, 13241-1-AP). Lane 1, yRD^−^:: GAL1p-Bax (lysate from untransformed cells, which do not contain CYP1A1 episomal plasmid, as negative control). Lanes 2-4: cell lysates from the strains yRD^+^, yRD^−^, and yRD^−^:: GAL1p-Bax transformed with an episomal plasmid that contains a CYP1A1 gene expression cassette under the control of the ADH2 promoter. Lane 2, yRD^+^:: CYP1A1, lane 3, yRD^−^:: CYP1A1, lane 4, yRD^−^:: Bax,CYP1A1, and lane 5, 1.5 pmole of microsomal CYP1A1 (as a positive control; CYP Design Ltd., Leicester, UK). (**b**) Lanes 2-4, same as in (**a**), the cell lysates being probed with an actin antibody (Proteintech, 60008-1-Ig). (**c**) Lanes 2-4, same as in (**a**), the cell lysates being probed with a c-myc antibody tagged to the human Bax protein (Thermo Scientific, Waltham, MA, USA, MA 1-980).

**Figure 6:**
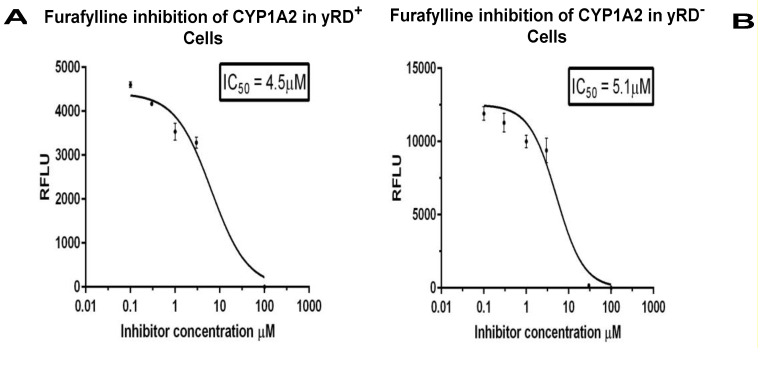

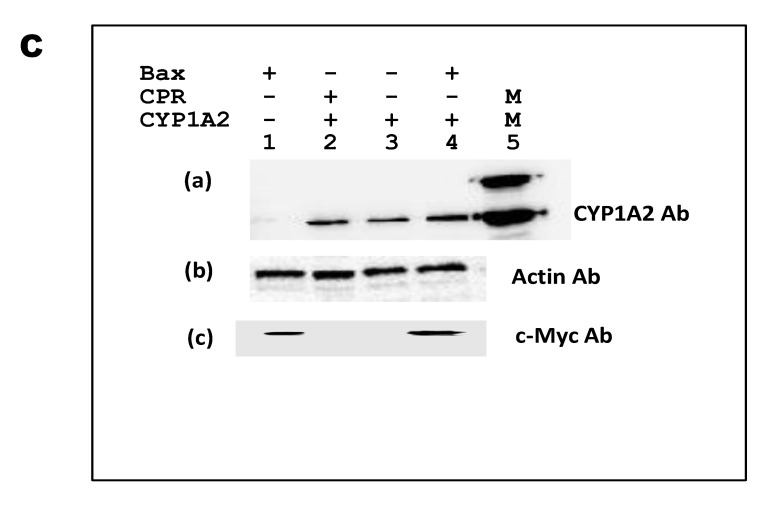
IC_50_ of Furafylline for the inhibition of CYP1A2 enzyme expressed within yRD^+^ and yRD^−^:: Bax cells. Furafylline’s IC_50_ for inhibition of CYP1A2 enzyme was expressed within whole cells. The cells that were used were from strains (**A**) yRD^+^ (+CPR) and (**B**) yRD^−^:: Bax (–CPR) were transformed with an episomal plasmid encoding a CYP1A2 gene expression cassette, CYP1A2 enzyme expression was driven by the ethanol-inducible ADH2 promoter (ADH2p). In yRD^−^ cells, Bax expression was driven by the GAL1 promoter (GAL1p), which was de-repressed in ethanol. Recombinant cells were grown for 18 h in YPD liquid medium containing glucose, allowing for around 6 h of induction and de-repression of ADH2p and GAL1p in ethanol (it takes ~12 h for glucose to be utilized and converted completely to ethanol). Cells were harvested and washed in PBS-saline. The assay included 1 × 10^7^ washed cells and 3-cyano-7-ethoxycoumarin (CEC) as a substrate. Each point represents the mean of triplicate readings; bars denote ± standard deviation. The *x*-axis represents the values of the concentration of furafylline in µM (ranges from 0.1 to 100 µM) that were incubated with cells for 10 min, the *y*-axis relative fluorescence units of the fluorescent product formed. The IC_50_ values were calculated using the GraphPad Prism software. Furafylline’s IC_50_ for inhibition of CYP1A2 Supersomes (insect cell-derived CYP1A2-bearing microsomes; Corning-Gentest), with a 10 min incubation with a microsomal enzyme, is 6.1 µM [36,37]. (**C**) Western Blotting of lysates from yRD^+^, yRD^−^, and yRD^−^:: Bax cells expressing CYP1A2. Yeast cell lysates (5 µg of total cellular protein for monitoring CYP1A2 and actin; 50 µg for Bax) fractionated on SDS/PAGE and probed with antibodies to CYP, Bax, and actin proteins after Western blotting. (aCell lysates probed with a CYP1A2 antibody (Proteintech, 19936-1-AP). Lane 1, BC600:: GAL1p-Bax (lysate from untransformed cells, which did not contain CYP1A2 episomal plasmid, as negative control). Lanes 2-4: cell lysates from the strains BC300, BC600, and BC600:: GAL1p-Bax transformed with an episomal plasmid that contained a CYP1A2 gene expression cassette under the control of the *ADH2* promoter. Lane 2, BC300:: CYP1A2, lane 3, BC600:: CYP1A2, lane 4, BC600:: Bax,CYP1A2, and lane 5, 1.5 pmole of microsomal CYP1A2 (as positive control; CYP Design Ltd.). (**b**) Lanes 2-4, as in (**a**), probed cell lysates with an actin antibody (Proteintech, 60008-1-Ig). (**c**) Lanes 2-4, same as in (**a**), the cell lysates being probed with a c-myc antibody tagged to the human Bax protein (Thermo Scientific, MA 1-980).

**Figure 7:**
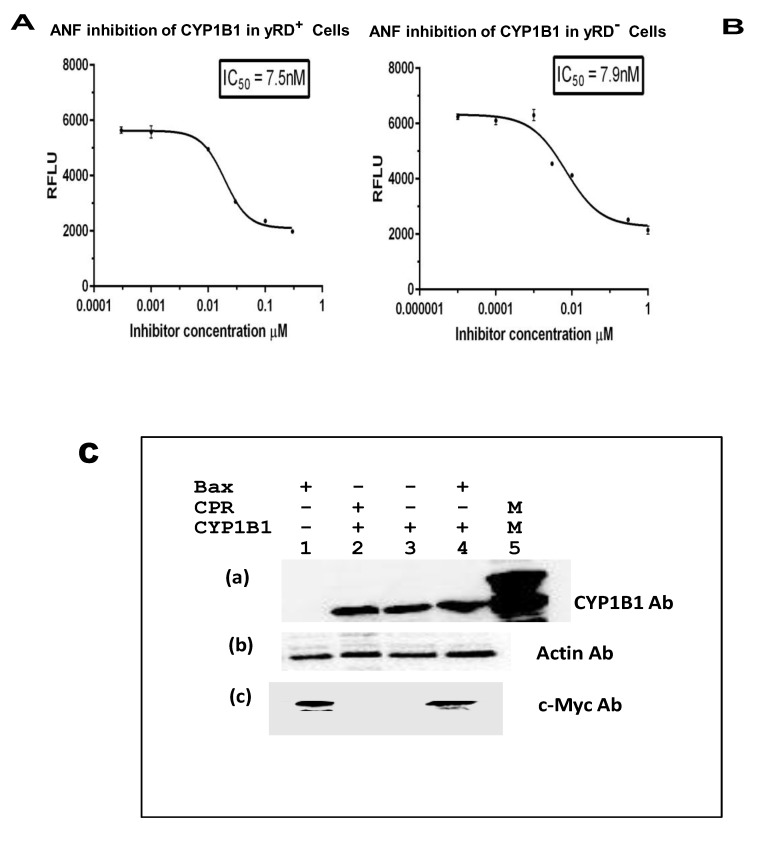
IC_50_ of α-naphthoflavone (ANF) for the inhibition of CYP1B1 enzyme expressed within yRD^+^ and yRD^−^:: Bax cells. ANF’s IC_50_ for inhibition of CYP1B1 enzyme that was expressed in live cells. The cells that were used were from strains (**A**) yRD^+^ (+CPR) and (**B**) yRD^−^:: Bax (–CPR). CYP1B1 enzyme expression was driven by the ethanol-inducible *ADH2* promoter (ADH2p). In yRD^−^ cells, Bax expression was driven by the *GAL1* promoter (GAL1p) which is de-repressed in ethanol. Recombinant cells were grown for 18 h in YPD liquid medium containing glucose, allowing for around 6 h of induction and de-repression of ADH2p and GAL1p in ethanol (it takes ~12 h for glucose to be utilized and entirely converted to ethanol). Cells were harvested, washed in PBS-saline. The assay included 1 × 10^7^ washed cells and 7-ethoxyresorufin (7-ER) as a substrate. The *x*-axis represents the values of the concentration of ANF in µM (ranges from 0.00001 to 3 µM) were incubated with cells for 10 min and the *y*-axis relative fluorescence units of the fluorescent product formed. The IC_50_ values were calculated using GraphPad Prism software. ANF’s IC_50_ for inhibition of CYP1B1 microsomes isolated from a lymphoblastoid cell line is reported to be 5 nM [38]. (**C**) Western Blotting of lysates from yRD^+^, yRD^−^, and yRD-:: Bax cells expressing CYP1B1. Yeast cell lysates (5 µg of total cellular protein for monitoring CYP1B1 and actin; 50 µg for Bax) fractionated on SDS/PAGE and probed with antibodies to CYP, Bax, and actin proteins after Western blotting. (**a**) Cell lysates probed with a CYP1B1 antibody (Proteintech, 18505-1-AP). Lane 1, yRD^−^:: GAL1p-Bax (lysate from untransformed cells, which do not contain CYP1B1 episomal plasmid, as negative control). Lanes 2-4: cell lysates from the strains yRD^+^, yRD^−^, and yRD^−^:: GAL1p-Bax transformed with an episomal plasmid that contains a CYP1B1 gene expression cassette under the control of the ADH2 promoter. Lane 2, yRD^+^:: CYP1B1, lane 3, yRD^−^:: CYP1B1, lane 4, yRD^−^:: Bax, CYP1B1, and lane 5, 1.5 pmole of microsomal CYP1B1 (as a positive control; CYP Design Ltd.). (**b**) Lanes 2-4, as in (**a**), probed cell lysates with an actin antibody (Proteintech, 60008-1-Ig). (cLanes 2-4, as in (**a**), probed cell lysates with a c-myc antibody tagged to the human Bax protein (Thermo Scientific, MA 1-980).

Alpha-naphthoflavone (ANF) is known to inhibit both CYP1A1 and CYP1B1 enzymes, as well as furafylline, a known inhibitor of CYP1A2. These were used for the IC_50_ determination studies (Figure 5A,B, Figure 6A,B, and Figure 7A,B). Western blotting (Figure 5C, Figure 6C and Figure 7C) was performed to confirm the expression of CYPs, driven by ADH2p, and Bax, driven by GAL1p, within yeast cells that lacked CPR activity. The presence of both a CYP and Bax further supported the notion that Bax indeed was responsible for the CYP activation. Although CYP-expressing yRD^−^ strains had CYP proteins as much as the cells that expressed CYPs in yRD^+^ and yRD^−^ strains, the protein was not active because of the absence of the P450 reductase (i.e., CPR).

## 4. Discussion

Results from this study demonstrated a possible communication between two sets of intracellular organelles: the mitochondria and the endoplasmic reticulum (ER). It was shown that inactive ER-bound human cytochrome P450 (CYP) enzymes, in the absence of their activating partner, the CYP450 reductase (CPR) (which is also normally bound to the ER membranes) can be re-activated by Bax, a pro-apoptotic human protein. This suggests that cytochrome P450 acts as a sink by sensing the generation of intracellular free electrons, eventually activating the inactive catalytic subunit. The ER is said to form a cloak around the mitochondria, and the mitochondria-ER interface is vital for this purpose [39]. The circumference of the mitochondria is surrounded by the ER, with mitochondrial constriction at the point of contact, followed by fission of the mitochondria [39]. ER proteins may play a part in the fission (i.e., division) of mitochondria. This suggests that electrons from the mitochondria, triggered by the association of Bax, can efficiently be utilized for CYP activation.

Figure 2A–C show the effects of CPR disruption on CYP1A2 activities in yRD^−^. As mentioned earlier, CPR is the primary electron transfer agent for CYPs. CPR delivers two electrons to the active site of the P450 haem in a highly organized process [40]. CYP activation usually occurs by allosteric binding of the reductase. Microsomal (i.e., endoplasmic reticulum-bound) CYPs need the reductase as a relay or intermediate to accept electrons from cellular NADPH and transport them consecutively (i.e., in a chain-like fashion) to the CYP active site. The importance of CPR cannot be over-emphasized. The results shown in Figure 2 reflect the essential nature of CPR and hoe it is crucial for CYP activity. CPR contains binding sites for FMN and FAD cofactors on its polypeptide chain. We revealed that NADP^+^ bound to the FAD site and electrons were serially delivered to a neighbouring FMN cofactor and finally to each CYP. Unusually, only one reductase aided the activity of all microsomal CYPs present in the human organism [40].

After having obtained the above results, further studies were carried out to assess if pro-apoptotic protein (Bax) served a role in the activation of inactive CYPs expressed in yRD^−^ devoid of CPR. The aim was to see if Bax, which naturally generates free electrons to produce ROS and thereby induce apoptosis, could compensate for the absence of CPR. Aiding the catalytic cycle and creating a biosensor environment was thought to possibly further develop a biosensor device. The comparison of CYP activities between yRD^+^ and yRD^−^ cells that express CYP1A2, irrespective of the promoter used, showed that there were significant differences in CYP1A2 activities between the two strains. To corroborate that CYP1A2 observations also apply to other CYPs, the ADH2 promoter was chosen for further studies with two more CYPs.

The results in Figure 3A–C show the differences in the CYP1A1, CYP1A2, and CYP1B1 activities in the three strains used to express yRD^+^ (+CPR), yRD^−^:: Bax (–CPR), and yRD^−^ (–CPR). The charts show that even after the disruption of yeast’s CPR, yRD^−^:: Bax significantly increased CYP activity as compared to the strain yRD^−^, which manifested nearly non-existent CYP activity. This would suggest that the presence of Bax compensated for the absence of CPR. These results indicated that Bax played a prominent role in compensating for the lack of reductase. In the early stages of inducing apoptosis, when the human Bax protein was mildly expressed under de-repressing conditions of the GAL1 promoter (i.e., in the presence of ethanol), it generated enough electrons to compensate for the absence of the P450 reductase (CPR), which was essential to transfer electrons to allow cytochrome P450 activity. Without a P450 reductase (CPR), a cytochrome P450 (CYP) was inactive. Electrons leaked from the mitochondria and this leakage was implicated as one of the factors responsible for ROS generation. According to Fisher (1988), electron leakages from the electron transfer chains were identified as a significant source of ROS within live cells. This was also observed in Figure 4, where the strains harbouring the Bax gene produced more ROS than others. It is well known that the mitochondrial respiratory chain produced substantial amounts of oxygen radicals (ROS) through oxidative phosphorylation. Electrons were carried through the respiratory chain to oxygen molecules (i.e., molecular oxygen, O2). This led to the generation/emission of protons, creating an electrochemical gradient [41]. ATP synthesis was driven by this gradient known as complex V.

When Bax was fully induced, yeast cells underwent apoptosis through ROS generation [42], Bax was a mitochondrial membrane-anchored protein, pro-apoptotic proteins may have the ability to generate electrons by creating pores on the mitochondrial membrane. These internally generated electrons reacted with oxygen to make ROS. The CYP450-CPR reaction also produces ROS superoxide, which was a reduced form of oxygen, and hydrogen peroxide, which was formed due to uncoupling the CYP450-CPR reaction [14]. Bax is a pro-apoptotic protein that translocates mitochondria from the cytosol and creates holes on the outer mitochondrial membrane during the process of apoptosis. This action aids the escape of more electrons from the mitochondria. The distribution and structure of mitochondria are controlled by fusion and fission events [39]. The mitochondria and the ER are strongly tied dynamic structures having all-encompassing contacts between them [39]. Mitochondrial fission happens at the point where the endoplasmic reticular tubules are in touch with the mitochondria which, in turn, mediate shrinking before recruitment of Drp1 protein, in mammals, or the homologue Dnm1 in yeast, which mediates fission [39].

The IC_50_ values of known inhibitors for the CYP enzymes expressed within recombinant cells (Figure 5, Figure 6 and Figure 7A) that co-express CYP and CPR (yRD^+^) and (Figure 5, Figure 6 and Figure 7B) co-express CYP and Bax in the absence of CPR are observed to be within the range. The values determined with these fluorescent assays on live cells within the range of values reported with CYP enzymes have been isolated from whole cells. This would indicate that Bax-activated CYPs expressed within cells could be used for high-throughput screening as an alternative for microsomal CYPs, which are difficult and expensive to produce. It is believed that electron transfer from microsomal cytochrome b5/NADH reductase (a second reductase system which is known to play a role in CYP activation in eukaryotic cells) is not enough to support on its own cytochrome P450 catalytic action. However, when cytochrome P450 reductase (CPR) gene is disrupted (i.e., deleted) in yeast (as it has been done for the yeast strain yRD^−^), microsomal cytochrome b5 may be able to supply the two electrons needed for activation of cytochrome P450 enzyme [43]. After disruption of the yRD gene in Saccharomyces cerevisiae, about 25% CPR (i.e., P450 reductase) activity was still seen [44]. Western blotting was also carried out to confirm the presence of CYP and Bax proteins in the respective strains (Figure 5, Figure 6 and Figure 7C)

**Figure 8:**
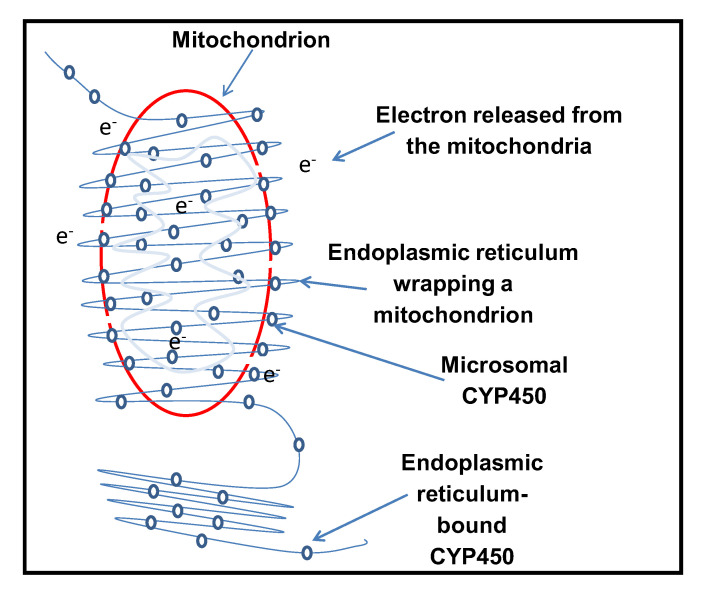
Represents a schematic diagram illustrating the possible activation of cytochrome P450 enzymes during minimal expression of Bax and also shows the potential communication between the mitochondria and endoplasmic reticulum (ER), displaying their strong ties and dynamics with their all-encompassing contact.

Electron transfer in biological systems occurred in the presence of electron donors such as NADPH or NADH, which was affected by electron transfer mediators (FAD and FMN). During reduction, NADPH and NADH were exhausted. One of the alternative methods of cytochrome P450 reduction gained ground as an electrochemical reduction with the help of an electrode as a direct electron source without any mediator. The supply of this electron from a non-natural source based on CYP P450 activation could form the basis of new biosensors, bioassays, and bioreactors. Mitochondria are not discrete organelles; they communicate with other organelles within the cell [41]. The mitochondria-ER contacts are features that are conserved in mitochondrial fission [39]. Mitochondrial fission regularly occurs at the point of interaction with the endoplasmic reticulum [39]. ER-mitochondrial communication seems to be essential for mitochondrial fission. However, stress can trigger mitochondrial hyper-fission, which can lead to the release of pro-apoptotic factors [45]. Figure 8 shows a schematic diagram illustrating the possible activation of cytochrome P450 enzymes and potential communication between the mitochondria and endoplasmic reticulum (ER).

Bax is a mitochondrial membrane-anchored protein that is pro-apoptotic. It is likely that proteins with an inherent ability to induce apoptosis can generate electrons that react with oxygen to produce ROS [42]. The useful improvement of haemoprotein-catalyzed reactions is essential. Electrodes have generally been used for CYP activation, which is associated with a CYP reductase (CPR) in the absence of NADPH [24]. Since both CPR and NADPH are incredibly relevant for CYP activation, the question was posed: can something else compensate for electron generation, required for CYP activation, which could substitute for CPR/NADPH? A protein that could spontaneously generate electrons within a live cell could substitute for the absence of CPR. Bax, being pro-apoptotic, destroys mitochondrial function by generating ROS [42]. It was conjectured that if the cells were to express a meagre amount of Bax, i.e., a level of protein that could not sufficiently induce apoptosis but still somewhat generate free electrons, it could mean that the absence of the reductase would be compensated. Low levels of Bax could experimentally be achieved by using an inducible promoter under conditions when the promoter was only de-repressed but not fully induced.

The presence of Bax was essential for inactive CYP enzyme activation, which was inactive because of the absence of CYP reductase (i.e., CPR) within the cells. CPR is critical for CYP activity. Bax’s presence most likely helped with the escape of electrons that were eventually used to activate a particular CYP. The contact or communication between mitochondria and the ER can itself create an environment for the release of pro-apoptotic factors, which potentially could activate inactive CYP enzymes. Yet this could not have resulted in CYP activation as the control strain, without Bax and CPR, did not show any activation. The supply of electron to the heme iron is essential for the catalytic cycle. This study investigated the development of CYP-based biosensors that are key to the emergence of a potential biocatalytic coupling system.

## 5. Conclusions

This study presented the development of a sensing system, which had a wide application. This could be developed into bioassays, bioreactors, or biosensors where biological molecules, i.e., CYP enzymes, could detect the presence of chemicals (substrates) for the screening of drugs by measuring biological or chemical reactions through the generation of signals in the cell. This suggested that the development of bioassays, bioreactors, and biosensors was possible using internally generated electrons to physiologically activate CYPs, which could be useful for research and disease diagnosis, among other things. This would enhance the routine use of CYP450s for purposes of high-throughput screening. Because CPR is toxic and NADPH is highly unstable, this system of generating electrons with the help of Bax would eliminate the complex electron transmission machinery involving the cytochrome P450 reductase (CPR). Advancement in biosensor development progression is pivotal for the improvement of an effective biocatalysts connection system, and this study is a step forward in developing a biosensor-based cytochrome P450. The above results show that three cytochrome P450 (CYP) isozymes, 1A1, 1A2, and 1B1, from the same CYP1 sub-family, were activated by Bax in the absence of P450 reductase (the yeast CPR, yRD). This alternative means of supplying electrons for CYP450 protein activation could lead to the generation of new systems, compatible and straightforward, in the development of methods and devices for the detection of xenobiotics, pollutants, drugs, which could lead to new therapeutic compounds.

## Supplementary Materials

Additional results. The following are available online at https://www.mdpi.com/1424-8220/20/14/4050/s1, Figure S1: The plasmid map of pRS305/GAL1p-h_Bax-MS showing restriction sites that cut the plasmid only once. The human Bax gene contains, at the 3’-end, a sequence that codes for the *c*-myc tag so that expressed protein can be monitored with an antibody that recognizes the *c*-myc epitope at the C-terminus of Bax protein. Figure S2: The three episomal plasmids used to introduce human CYP gene expression cassettes, under the control of the *ADH2* promoter. (**A**) pSYE266, a yeast expression plasmid that bears the CYP1B1 gene driven by the ADH2 promoter; (**B**) pSYE271, a yeast expression plasmid that bears the CYP1A1 gene driven by the ADH2 promoter; (**C**) pSYE265 a yeast expression plasmid that bears the CYP1A2 gene driven by the ADH2 promoter. The restriction sites shown occur only once in the plasmid. Figure S3: Map of plasmid pAUR101, showing only the restriction sites that were used for constructing and cloning of the ΔyRD (delta-yRD) gene insert. Figure S4: Strategy for construction of the integrating plasmid pAUR101/ΔyRD. Plasmid pSP73/yRD (plasmid’s insert is shown in (**A**)) was digested with MunI and re-ligated to construct the plasmid pSP73/ΔyRD(a) (plasmid’s insert is shown in (**B**)). In the process, 682 bp from the middle of yRD gene was deleted, out-of-frame, to obtain the truncated ΔyRD(a) gene (B). A further 115 bp was deleted from the 5’-end of ΔyRD(a) gene to obtain an EcoRV-SalI fragment of ΔyRD (**C**) which was cloned at the SmaI, SalI sites of pAUR101 to obtain the plasmid pAUR101/ΔyRD (**D**). Multiple restriction enzyme digestions confirmed the authenticity of the plasmid. The map shows only the restriction sites that were used for constructing and cloning of the ΔyRD (delta-yRD) gene insert. The SwaI site was used for linearizing the plasmid for gene disruption at yRD’s endogenous locus.
