# Supplementary material for: Sensing the Generation of Intracellular Free Electrons Using the Inactive Catalytic Subunit of Cytochrome P450s as a Sink
Source: Sensors (Basel). 2020 Jul 21;20(14):4050. doi: 10.3390/s20144050 (PMC7411652; doi:10.3390/s20144050)
Supplement: Supplementary file 1 [file sensors-20-04050-s001.pdf]

sensors

MDPI

Supplementary materials

Sensing the Generation of Intracellular Free Electrons Using the Inactive Catalytic Subunit of Cytochrome P450s as a Sink

Damilare D. Akintade [1,2,*] and Bhabatosh Chaudhuri [2]

1 School of Life Sciences, Medical School, University of Nottingham, Nottingham NG7 2UH, UK
2 Leicester School of Pharmacy, De Montfort University, Leicester LE1 9BH, UK; BChaudhuri@dmu.ac.uk
* Correspondence: Damilare.Akintade@nottingham.ac.uk; Tel: +44-07712452922

Commented [M1]: Newly added information, please confirm.

Commented [DA2R1]: Thant's fine

Commented [M3]: Newly added information, please confirm.

Commented [DA4R3]: That's fine

1. The plasmid construct bearing the human Bax-α gene and the resultant yeast strain

The human *Bax-α* (*Bax*) gene is under the control of the galactose-inducible *GAL1* promoter and was cloned in an integrating vector that bears the *LEU2* auxotrophic marker. The resultant plasmid was integrated into the *LEU2* chromosomal locus of the yeast strain yRD^{-}. A control plasmid, which does not contain any gene insert, was also integrated into the *LEU2* chromosomal locus of yRD^{-}. The map of the plasmid are shown below.

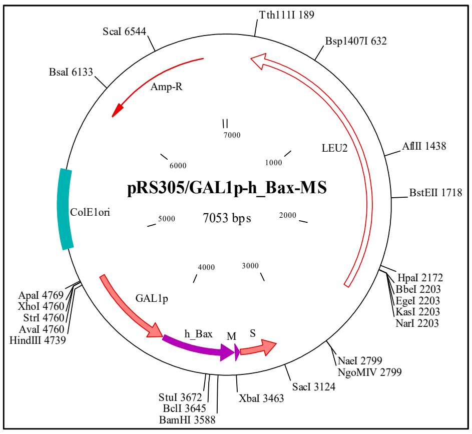

Figure S1. The plasmid map of pRS305/GAL1p-h_Bax-MS showing restriction sites that cut the plasmid only once. The human Bax gene contains, at the 3'-end, a sequence that codes for the *c*-myc tag so that expressed protein can be monitored with an antibody that recognizes the *c*-myc epitope at the C-terminus of Bax protein.

2. Plasmid constructs bearing the human Cytochrome P450 genes (CYP1A1, CYP1A2, and CYP1B1) and the resultant yeast strains

The human cytochrome P450 genes (CYP1A1, CYP1A2, and CYP1B1) were cloned downstream of the ADH2, GAPDH, or PGK1 promoter in episomal plasmids bearing URA3 auxotrophic marker. The plasmids were transformed into the yeast strains yRD^{+} and yRD^{-}. The plasmid maps are shown below.

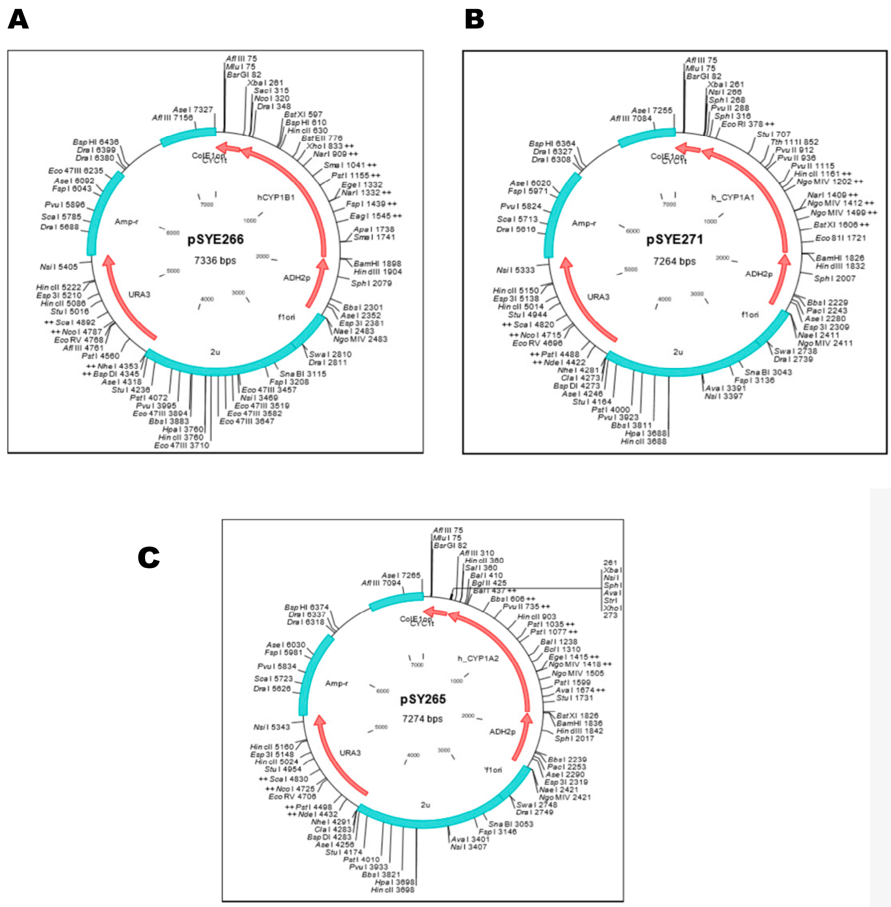

Figure S2. The three episomal plasmids used to introduce human CYP gene expression cassettes, under the control of the *ADH2* promoter. (**A**) pSYE266, a yeast expression plasmid that bears the CYP1B1 gene driven by the ADH2 promoter; (**B**) pSYE271, a yeast expression plasmid that bears the CYP1A1 gene driven by the ADH2 promoter; (**C**) pSYE265 a yeast expression plasmid that bears the CYP1A2 gene driven by the ADH2 promoter. The restriction sites shown occur only once in the plasmid.

3. Creation of CPR-null Yeast cells from CPR-plus Yeast cells (W303-A1). (Deletion of the yeast endogenous NADPH reductase (yRD) from the yeast strain (W303-A1), using gene disruption technology, to obtain the strain BC300 yrd^{-})

In gene disruption protocols, yeast transformants (i.e., colonies obtained after transformation of DNA into yeast) are sometimes selected using a drug resistance gene as a marker [1,2]. The main drawback in using antibiotic resistance markers in yeast molecular biology is the fact that yeast is quite resistant to most antibiotics. The yeast endogenous reductase gene (yRD) in the yeast strain yRD^{+} has been disrupted using the *aureobasidin A* (AbA) resistance gene as a selectable marker.

Aureobasidin, an antifungal antibiotic produced by Aureobasidium pullulan, is very toxic to the budding yeast S. cerevisiae and also the fission yeast S. pombe. The plasmid pAUR101 is an

integrating chromosomal vector that allows integration of a gene of choice into a chromosomal locus of S. cerevisiae. This vector does not replicate autonomously in yeast but is maintained in yeast cells only when integrated into the chromosome by recombination. The plasmid pAUR101 has a novel drug-resistant selection marker, AUR1-C gene, which is a mutant gene derived from the S. cerevisiae genome and confers aureobasidin A-resistance on yeast cells. The plasmid pAUR101was purchased from Takara (Takara, Japan, Cat. No. 3600); the plasmid is depicted in Figure S3.

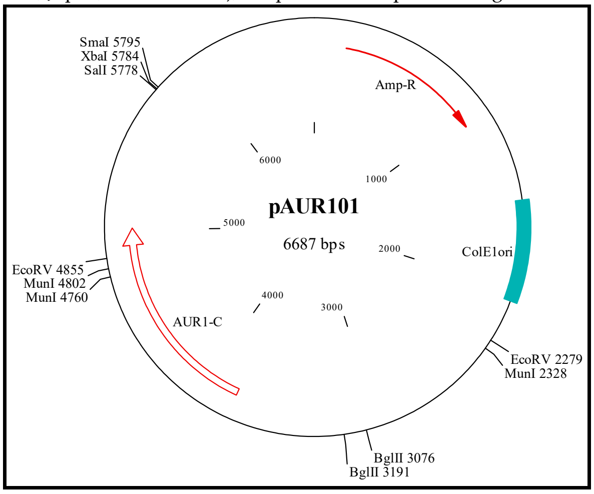

Figure S3. Map of plasmid pAUR101, showing only the restriction sites that were used for constructing and cloning of the ΔyRD (delta-yRD) gene insert.

To disrupt the yeast endogenous P450 reductase, yRD, using the pAUR101 plasmid, a DNA fragment that contained (a) a 682 bp deletion from the middle of the *yRD* gene and (b) a 115 bp deletion at the 5′-end of *yRD* was inserted into the pAUR101 plasmid at the *Sma*I, *Sal*I sites. This yielded a new plasmid which was named pAUR101/ΔyRD. It was cut at the unique *Swa*I site, within the *ΔyRD* genetic sequence (Figure S4); the linearized fragment was introduced into the genome of the yeast strain yRD^+ via homologous recombination to obtain the strain yRD^-. This strain played an essential part in the experiments performed in the Manuscript. It helped in clearly showing that completely independent of the endogenous yeast P450 reductase (yRD) or any other P450 reductase, pro-apoptotic human protein Bax can activate inactive human CYPs.

To obtain the plasmid pAUR101/ΔyRD, the plasmid pSP73/yRD, containing the full-length *yRD* gene, was at first completely digested with *Mun*I and then re-ligated to delete a 682 bp fragment from the middle of the yeast reductase gene to yield a new plasmid designated pSP73/ΔyRD(a). The *ΔyRD(a)* DNA fragment was isolated as an *Eco*RV-*Sal*I fragment (this deletes 115 bp from the 5′-end) and this fragment was inserted into pAUR101 at the *Sma*I, *Sal*I sites to construct a new plasmid designated as pAUR101/ΔyRD. The strategy used for the construction of pAUR101/ΔyRD is shown in Figure S4.

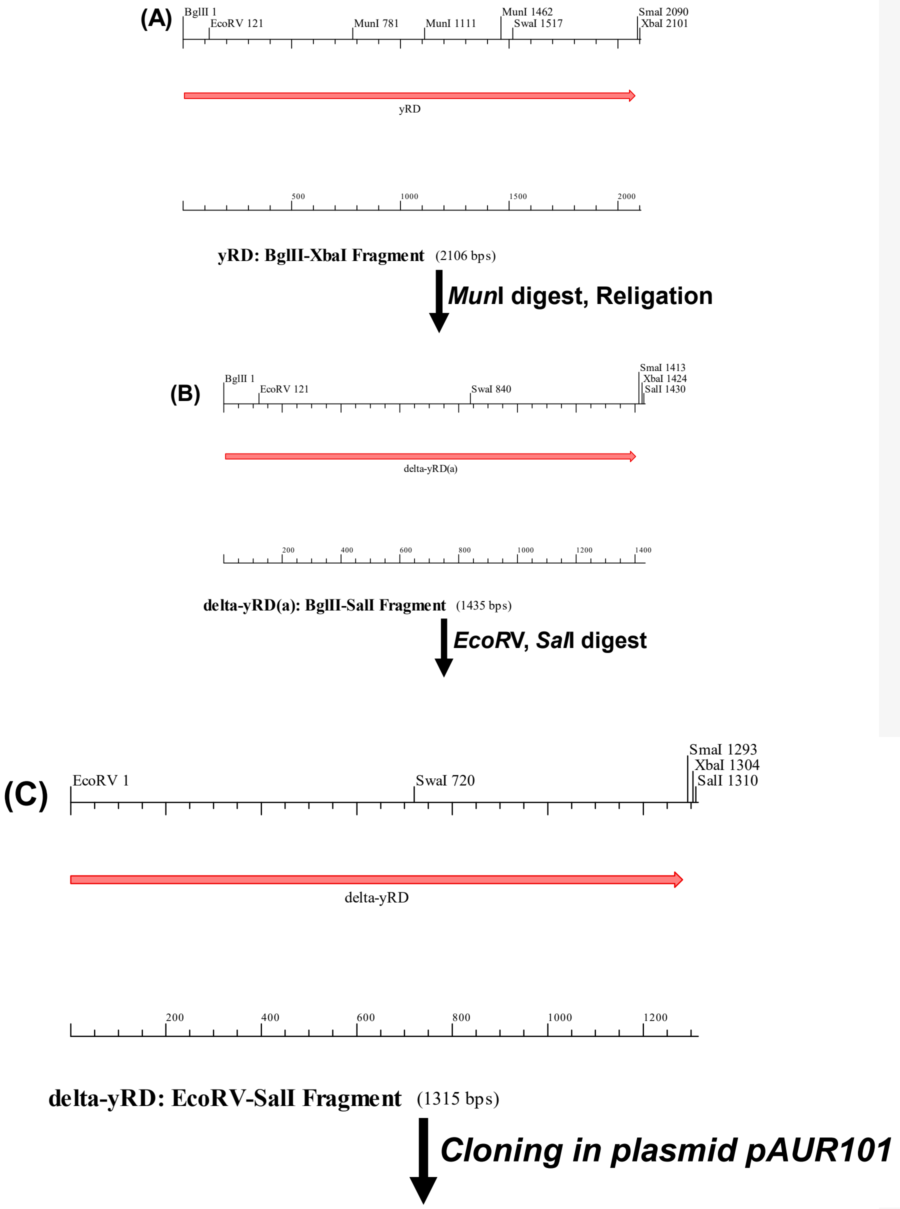
(A)
BglII 1
EcoRV 121
MunI 781
MunI 1111
MunI 1462
SwaI 1517
SmaI 2090
XbaI 2101
yRD
500
1000
1500
2000
yRD: BglII-XbaI Fragment (2106 bps)
MunI digest, Religation
(B)
BglII 1
EcoRV 121
SwaI 840
SmaI 1413
XbaI 1424
SalI 1430
delta-yRD(a)
200
400
600
800
1000
1200
1400
delta-yRD(a): BglII-SalI Fragment (1435 bps)
EcoRV, SalI digest
(C)
EcoRV 1
SwaI 720
SmaI 1293
XbaI 1304
SalI 1310
delta-yRD
200
400
600
800
1000
1200
delta-yRD: EcoRV-SalI Fragment (1315 bps)
Cloning in plasmid pAUR101

(D)

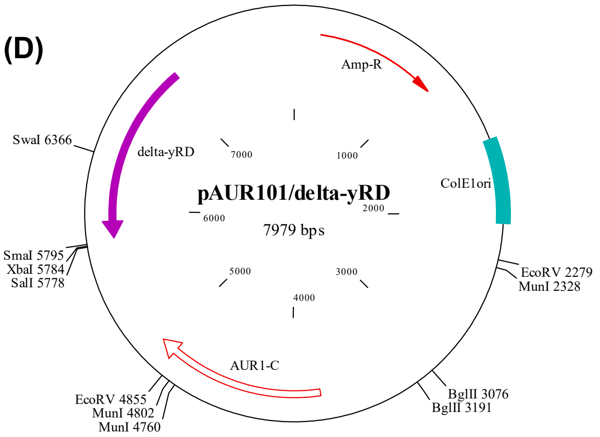

Figure S4. Strategy for construction of the integration plasmid pAUR101/ΔyRD. Plasmid pSP73/yRD (plasmid's insert is shown in (**A**)) was digested with MunI and re-ligated to construct the plasmid pSP73/ΔyRD(a) (plasmid's insert is shown in (**B**)). In the process, 682 bp from the middle of yRD gene was deleted, out-of-frame, to obtain the truncated ΔyRD(a) gene (B). A further 115 bp was deleted from the 5′-end of ΔyRD(a) gene to obtain an EcoRV-SalI fragment of ΔyRD (**C**) which was cloned at the SmaI, SalI sites of pAUR101 to obtain the plasmid pAUR101/ΔyRD (**D**). Multiple restriction enzyme digestions confirmed the authenticity of the plasmid. The map shows only the restriction sites that were used for constructing and cloning of the ΔyRD (delta-yRD) gene insert. The SwaI site was used for linearizing the plasmid for gene disruption at yRD's endogenous locus.

Commented [M5]: Please replace with a sharper image.

Finally, in order to disrupt the yRD gene of S. cerevisiae, the plasmid pAUR101/ΔyRD (Figure S4 (D)) was linearised by cutting at the unique SwaI site. The resulting linearised DNA was then introduced into the yRD$^+$ yeast strain. The integrants were selected on SD plates containing 0.5 µg/µl of aureobasidin (Takara, Japan, Cat. No. 9000).

References

1. Hashida-Okado, T.; Ogawa, A.; Kato, I.; Takesako, K. Transformation system for prototrophic industrial yeasts using the AUR1 gene as a dominant selection marker. *FEBS Lett.* **1998**, *425*, 117–122.
2. Ogawa, A.; Hashida-Okado, T.; Endo, M.; Yoshioka, H.; Tsuruo, T.; Takesako, K.; et al. Role of ABC transporters in aureobasidin A resistance. *Antimicrob. Agents Chemother.* **1998**, *42*, 755–761.
